# Flow measurement by cardiovascular magnetic resonance: a multi-centre multi-vendor study of background phase offset errors that can compromise the accuracy of derived regurgitant or shunt flow measurements

**DOI:** 10.1186/1532-429X-12-5

**Published:** 2010-01-14

**Authors:** Peter D Gatehouse, Marijn P Rolf, Martin J Graves, Mark BM Hofman, John Totman, Beat Werner, Rebecca A Quest, Yingmin Liu, Jochen von Spiczak, Matthias Dieringer, David N Firmin, Albert van Rossum, Massimo Lombardi, Juerg Schwitter, Jeanette Schulz-Menger, Philip J Kilner

**Affiliations:** 1 CMR Unit, Royal Brompton Hospital, London, UK; 2 Department of Physics and Medical Technology, VU University Medical Center, Amsterdam, the Netherlands; 3 University Department of Radiology, Addenbrooke's Hospital, Cambridge, UK; 4 Division of Imaging Sciences, King's College, London, UK; 5 Division of Neuroradiology and Magnetic Resonance, University Children's Hospital, Zurich, Switzerland; 6 Radiological Sciences Unit, The Hammersmith Hospitals NHS Trust, London, UK; 7 Auckland MRI Research Group, University of Auckland, Auckland, New Zealand; 8 Institute for Biomedical Engineering, University and ETH Zurich, Zurich, Switzerland; 9 Franz-Volhard-Klinik, Charité Universitätsmedizin, Berlin, Germany; 10 Department of Cardiology, VU University Medical Center, Amsterdam, the Netherlands; 11 Magnetic Resonance Laboratory, Italian National Research Council (CNR), Pisa, Italy; 12 Cardiac MRI Center, University Hospital Zurich, Zurich, Switzerland; 13 Franz-Volhard-Klinik, Charité Universitätsmedizin, Berlin, Germany

## Abstract

**Aims:**

Cardiovascular magnetic resonance (CMR) allows non-invasive phase contrast measurements of flow through planes transecting large vessels. However, some clinically valuable applications are highly sensitive to errors caused by small offsets of measured velocities if these are not adequately corrected, for example by the use of static tissue or static phantom correction of the offset error. We studied the severity of uncorrected velocity offset errors across sites and CMR systems.

**Methods and Results:**

In a multi-centre, multi-vendor study, breath-hold through-plane retrospectively ECG-gated phase contrast acquisitions, as are used clinically for aortic and pulmonary flow measurement, were applied to static gelatin phantoms in twelve 1.5 T CMR systems, using a velocity encoding range of 150 cm/s. No post-processing corrections of offsets were implemented. The greatest uncorrected velocity offset, taken as an average over a 'great vessel' region (30 mm diameter) located up to 70 mm in-plane distance from the magnet isocenter, ranged from 0.4 cm/s to 4.9 cm/s. It averaged 2.7 cm/s over all the planes and systems. By theoretical calculation, a velocity offset error of 0.6 cm/s (representing just 0.4% of a 150 cm/s velocity encoding range) is barely acceptable, potentially causing about 5% miscalculation of cardiac output and up to 10% error in shunt measurement.

**Conclusion:**

In the absence of hardware or software upgrades able to reduce phase offset errors, all the systems tested appeared to require post-acquisition correction to achieve consistently reliable breath-hold measurements of flow. The effectiveness of offset correction software will still need testing with respect to clinical flow acquisitions.

## Introduction

Phase contrast cardiovascular magnetic resonance (CMR) [1] measurements of flow through planes transecting the great arteries are used clinically for calculations of cardiac output, shunt flow [2,3] or aortic or pulmonary regurgitation [4,5]. In combination with measurements of left ventricular volume or mitral inflow, measurement of aortic outflow may also allow the indirect calculation of mitral regurgitation [5-7]. Such measurements are non-invasive and require no contrast agent or ionising radiation. They represent a capability unique to CMR which can be of considerable value in clinical investigation and research. However, the derivation of cardiac output, regurgitant or shunt flow from velocity images calls for a very high standard of accuracy, requiring the minimisation of background phase offset errors, which are the focus of this paper. Note that this paper examines the offsets before any correction technique has been applied, such as static tissue or static phantom baseline correction, which may generally reduce the problem subject to the reliability of the correction method itself.

**Figure 1:**
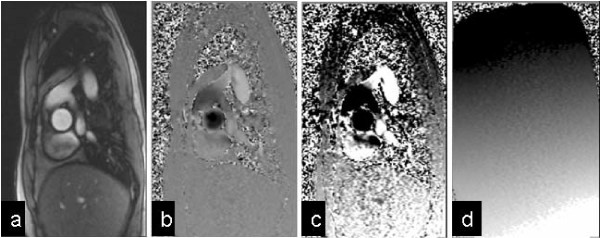
**A systolic frame of an aortic flow acquisition**. (170 ms after R-wave, at Venc = 150 cm/s). (a) Signal magnitude image, (b) Phase contrast velocity image shown at normal greyscale settings (black = -150 cm/s, white = +150 cm/s) where there apparently uniformly grey chest wall fails to reveal the background offset error. The same image is therefore reprinted in (c) with more extreme greyscale contrast to show up the background offset errors (black ≤ -15 cm/s, white ≥ +15 cm/s) (d) Phase contrast image using identical sequence protocol, but of static gelatin phantom, displayed with same greyscale as (c), demonstrating the phase offset.

**Figure 2:**
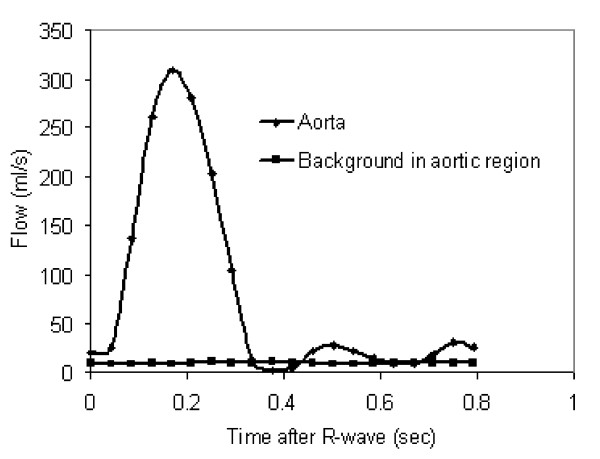
**Aortic flow 64 ml/beat measured from Figure 1**. The background in the aortic region was measured in the phantom, as in Figure 1d. The aortic flow curve includes 8.4 ml/beat due to the background offset of 1.6 cm/s in the aortic region. The true aortic flow is 56 ml/beat. The relative error in the calculated flow measurement is therefore 15%. Although the example in Figure 1 may be relatively easy to correct by correcting phase offset errors of signal across the relatively large regions of static chest wall and liver, correction is not always as straightforward in clinical acquisitions. Without such an independent correction of the background offset, it would be difficult to correct the aortic flow curve by using physiological assumptions such as negligible flow in diastole.

As illustrated in Figure 1, clinical flow acquisitions can be subject to small positive or negative phase offset errors. They can be recognised where stationary tissue shows small apparent velocities which tend to increase with distance from the centre of the image. Phase offset errors may involve regions of flow measurement. They may vary unpredictably with slice orientation, slice shift (along the slice-select direction) and with other parameters that affect the gradient waveforms or their timings. They generally vary gradually with position over the image and are stable over all frames of a properly retro-gated cine. Although typically small, of the order of 1 or 2 cm/s, they matter because calculations of volume flow are based on the summation of velocities through the whole cross sectional areas of vessels and also through all phases of the cardiac cycle. Because of these two summations, the small background velocity offset error accumulates to give potentially significant errors in the calculated volume flow (Figure 2).

The background offset errors in typical cardiac flow applications have been studied previously e.g. [8-10] and their consequences can be estimated as follows. For example, consider a 5% error in a stroke volume of 80 ml/beat, which is 4 ml, which we suggest may represent a limit of acceptability. If this were measured over a great vessel of diameter 30 mm and through an R-R interval of 1 second, the 4 ml error could result from a mean velocity error of only 0.57 cm/s. This velocity offset corresponds to less than 0.4% of a typical velocity-encoding range (Venc) of 150 cm/s (or 0.3% of 200 cm/s). The high sensitivity of derived flow measurement to small errors in velocity is attributable to the double summation, over the vessel area and throughout the R-R interval. Given only 0.6 cm/s offset errors, as above, the calculation of shunt flow from the difference between pulmonary and aortic flow measurements might be affected by up to 10%, if the background errors were to have opposing polarities in the two acquisitions [9]. The effects on measurements of valve regurgitation are harder to summarise. Considering a regurgitant fraction (RF) of 15% as an example, corresponding to a 15 ml reverse flow during 600 ms of diastole after 100 ml of forward flow during 400 ms of systole, the 0.6 cm/s offset discussed above would cause the RF to be miscalculated as either 12.5% or 17.5%, depending on the polarity of the offset. Relative to moderate and severe regurgitation the error in RF may appear less because the offset results in a smaller relative miscalculation of the larger reverse flow, although the reduction would partially be cancelled by the increased velocity encoding range that would be needed to avoid aliasing during the increased amount of forward flow. Please note that the estimates above are based on a velocity offset of 0.6 cm/s, which we propose as a theoretical limit of acceptability.

When baseline offset correction methods have been applied with proof of their *in-vivo *efficacy [9,10], the resulting CMR measurements of flow have been found to have a high accuracy, which may be hard to achieve by any other *in-vivo *modality. It should be understood that this study uses uncorrected offset data, revealing how much dependence there is on the correction methods and their routine *in-vivo *reliability.

The study reported here was initiated by members (PJK, JS, AvR, JS-M, ML) of the EuroCMR Working Group of the European Society of Cardiology (ESC). Among them, they had experience of several types of commercially available CMR system, and they shared concerns regarding possible inaccuracies in derived flow measurements. They agreed that background phase offset errors were, amongst other possible problems, likely to be the principal cause for concern. Therefore, the purpose of this study was to use static gel phantoms to investigate whether their concerns were justified, potentially motivating further optimisation of CMR velocity mapping for clinical flow measurements. The ability of CMR to rapidly and non-invasively measure flow through planes transecting the large vessels is unique, clinically valuable and worth optimising.

## Methods

### Static gel phantom

To eliminate the possibility of convection or motion-induced currents within fluid phantoms, an aqueous solution of gelatine was set within 10-15 litre uniform plastic tanks, with sufficient dimensions at all sites to enable measurement over the regions specified below. To reduce T1 for improved signal to noise ratio of the gelatine, 5 mmol/l of Gd-DTPA was added. These methods were adopted because the background offsets being studied were potentially very small.

### CMR Systems tested

The study was limited to 1.5 T as this is currently the most widely-used main field strength for CMR. Automatic correction of concomitant gradient terms [11] was employed, whereas any other filtering or correction of background offset errors was turned off. Only CMR systems with higher gradient performance supporting breath-hold flow imaging within the range of imaging parameters specified below were included. Three 1.5 T scanner types were used, one from each of three manufacturers. We acquired static phantom phase offset datasets using twelve separate 1.5 Tesla CMR systems, four each of the three different types (See Acknowledgements section; this change was required by the publisher in final proofreading for some mysterious reason).

### Phase contrast velocity acquisitions

To ensure consistent test protocols for each type of scanner, four of the investigators (PG, MPR, MJG, JT) set up an acquisition protocol for each type of system, and transferred this protocol to the other sites of that type. This protocol included the slice orientations described below.

We aimed to use similar phase-contrast sequence parameters for each of the three scanner types. The following sequence parameters were reproduced for each type (merely as a model for a typical clinical exam and not necessarily representing a recommendation of a set of optimized parameters for a breath-hold through-plane flow study). All cine phase-contrast acquisitions were by retrospectively gated pulse sequences, where the phase-encode was updated by each detected ECG R-wave. The continuous gradient activity of this approach, with no silent gap while waiting for the next R-wave, has the advantage of a more stable background offset during the cardiac cycle [12], as well as enabling late diastolic imaging. All acquisitions used an ECG simulator at 1 second R-R interval, through-plane velocity-encoding at Venc = 150 cm/s, slice thickness 6 mm, FOV = 320 mm square, uninterpolated pixels 1.25 mm(FE) by 2.5 mm(PE), flip angle 22°, 6 raw data lines per cardiac cycle, and no parallel imaging. These parameters often required "first-level" operation of the gradient system with respect to peripheral nerve stimulation. The square FOV is of course atypical for cardiac work, but was adopted to avoid centres modifying FOVs to avoid PE-wraparound of the large phantoms sometimes used. Similarly unusual, two averages were used to ensure adequate SNR for measurement of the small velocity offsets. Neither of these adaptations would be expected to modify the background offset error. Certain other aspects of the pulse sequence were beyond our control using standard clinical sequences, and these are listed below for each type of scanner. Unless stated below, the velocity encoding was asymmetric (i.e. it used phase-subtraction of velocity-compensated and velocity-encoded sequence repetitions). For all of the sequences, the gradient-echo was asymmetric (i.e. the gradient-echo rephased early in the ADC sampling window for short TE). The TR values stated were between the RF excitation pulses, and in all cases the true flow cine temporal resolution was 12 × TR. A larger number of *temporally interpolated *cine phases was reconstructed by the Siemens and GE machines, but without consequences for the true flow acquisition.

Systems used were as follows:

Philips Achieva R2.53 (4 sites). TR5.5 ms, TE2.8 ms, pixel bandwidth 355 Hz/pixel (Fat/Water Shift 0.62). The slice-selective RF pulse used an asymmetric design with a late centre. The background phase-offset correction ("LPC filter") was switched off for this study (see Discussion). Fifteen cardiac phases were reconstructed (*i.e*. temporal interpolation was not performed during reconstruction).

Siemens Avanto VB15 TR6.6 ms (4 systems over 3 sites), TE2.8 ms, sampling bandwidth 355 Hz/pixel. The controls for RF pulse and gradient mode, which control the use of faster and stronger RF and gradient pulses, were both set to "Normal" mode in order to achieve TR and TE similar to the other scanners. Twenty-five cardiac phases were reconstructed (*i.e*. temporal interpolation was applied by reconstruction).

GE Signa Excite 14M5 (4 systems over 3 sites). This used symmetric velocity-encoding (i.e. two sequence repetitions with positive and negative velocity sensitivities around the velocity-compensated waveform, also known as "balanced" velocity-encoding). The "flow analysis" flag was on, disabling a spatial high-pass filter used for phase-contrast angiography background suppression. (In a slightly different form this filter apparently resembles the Philips approach to background correction). The GE "flow optimization" control resulted in longer TE and TR than the other scanners and was therefore not used. The readout ADC bandwidth was 41.67 kHz (pixel bandwidth 326 Hz/pixel). On the GE, the TR and TE ranged over 5.9-6.0 ms and 2.9-3.0 ms respectively for the oblique slices tested but were reproduced exactly at all 4 sites, as the sequence optimised its timings depending on image plane orientation. Twenty cardiac phases were reconstructed (*i.e*. temporal interpolation was applied by reconstruction).

**Figure 3:**
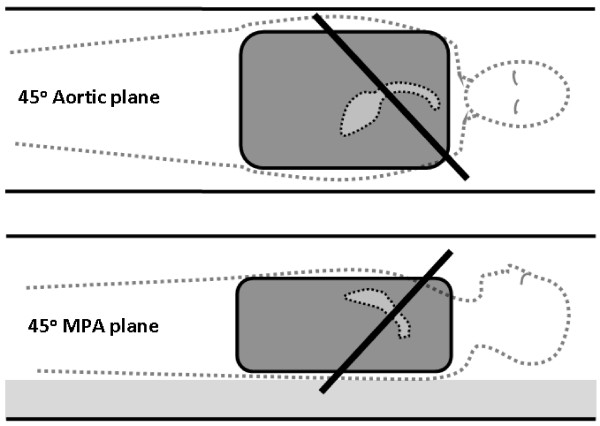
**Coronal (upper) and sagittal (lower) illustrations of the oblique planes of velocity acquisition (thick black lines)**. The Aortic plane, for which an antero-posterior phase-encode direction was always chosen, was at 45° between transverse and sagittal. The main pulmonary artery (MPA) plane, which was acquired twice, either with left-right or head-foot phase-encode directions, was at 45° between transverse and coronal. Each plane passed through the isocentre. The dark grey region represents the uniform gelatin phantom, with dotted lines indicating a corresponding patient position with typical orientations of the aorta and MPA in which flows are typically measured through slices similar to those of this study.

Each centre acquired the same two planes (3 acquisitions), as defined in the transferred protocol files (Figure 3). Again, we emphasise that these were defined for scanner comparison rather than making any form of recommendation. The 'aortic' (Ao) plane was 45° oblique between transverse and sagittal planes, with anterior-posterior phase-encoding. The 'main pulmonary artery' (MPA-LR and MPA-HF) plane was 45° oblique between transverse and coronal planes, the two acquisitions using left-right and head-foot phase-encoding, respectively. All three acquisitions were centered on the isocentre.

### Analysis of phantom 'velocity' map data

**Figure 4:**
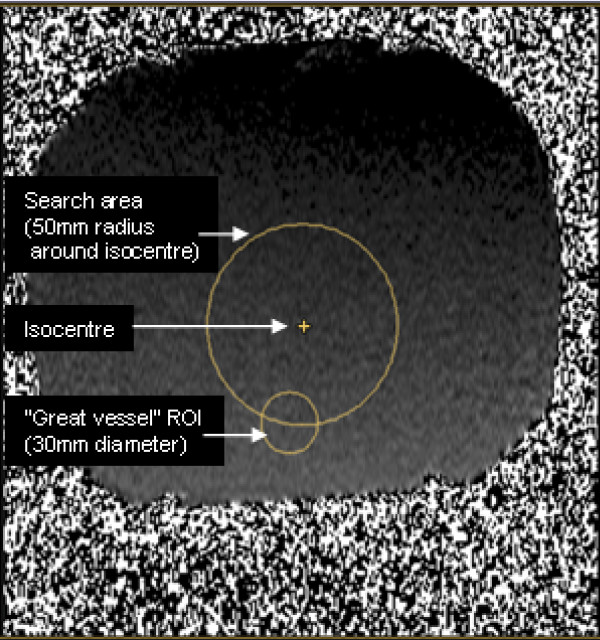
**The flow offset analysis method**. The mean phase offset over the smaller 30 mm diameter circle, which represents a typical "great vessel" ROI, was calculated for all positions of such ROIs with their centres located up to 50 mm from the isocentre of the magnet (up to 70 mm for MPA planes). The largest ROI value found was taken as the 'worst case' result for that plane of acquisition. All image planes passed through the isocentre. In this example, the largest error was found in the mean ROI positioned at the lower edge of the search region.

All images were analyzed independently by two sites using independent software written at each site in Matlab (The MathWorks, Natick, USA). The software reported the most extreme mean velocity offset measured in a circular 'great vessel' region, 30 mm diameter [13], centered within specified distances (see below) from the centre of each velocity map. It aimed to record the 'worst-case' error that might affect typical great vessel flow measurements. The relevant extent of regions interrogated was estimated from the locations of the ascending aorta and MPA in a survey of clinical flow studies. For the Ao plane the maximum distance was set at 50 mm from the image centre (Figure 4) and for both MPA planes, 70 mm from the image centre, which was located at the magnet isocentre in all cases. The larger span of regions for MPA analysis reflected the more anterior location of the MPA in patient studies. Working on the averaged image of all the frames of the cine, the largest mean apparent velocity in cm/s found by any of the 30 mm diameter circular regions centred within the defined spatial limits was recorded for each plane studied. The result reported was temporally averaged over all of the cine frames. The uncertainty in the results was assessed using the standard deviation across the cine frames (i.e. temporal rather than spatial standard deviation) of the region's mean value. The study did not aim to compare the signal to noise ratio of velocity measurements between systems. The uncertainty was a combination of random noise of any frame-to-frame variations during the retro-gated velocity cine, and was stated as an indicator of the offset assessment's reliability.

## Results

Table 1 shows the average of the two independent analyses, for all three planes at all 12 scanners. As an overall indication of the amount of offset, the average, ignoring the polarity of the offset, over all 12 scanners for the Aortic, MPA(head-foot phase-encoding) and MPA(left-right phase-encoding) planes gave 1.6 cm/s, 2.9 cm/s and 3.7 cm/s, respectively. The results of the two image analyses agreed with an uncertainty range of less than ± 0.2 cm/s, as defined above.

**Table 1:** Largest mean uncorrected velocity offset (cm/s) in regions centred within 50 mm of isocentre for the aortic plane, and within 70 mm for the MPA plane (head-foot or left-right phase-encoding).

cm/s	Scanner Site	Aorta	MPA (HF phase-enc)	MPA (LR phase-enc)
Scanner type 1	1	-2.2 cm/s	-3.8 cm/s	-5.6 cm/s

	2	-2.8	-3.1	-5.5

	3	-0.7	-3.2	-5.3

	4	-1.3	-3.5	-4.7

Scanner type 2	5	-2.2	-3.4	-5.4

	6	-1.1	-3.9	-3.1

	7	1.6	-4.0	-3.6

	8	-2.7	-4.9	-4.9

Scanner type 3	9	1.6	1.2	1.8

	10	1.2	-0.7	0.4

	11	0.9	1.6	2.1

	12	1.1	-1.7	2.0

Mean of all 12 absolute values		1.6 cm/s	2.9 cm/s	3.7 cm/s

Range of all 12 absolute values		0.7 to 2.8 cm/s	0.7 to 4.9 cm/s	0.4 to 5.6 cm/s

The twelve rows are from the four sites using each scanner type. The scanner types are not identified.

**Figure 5:**
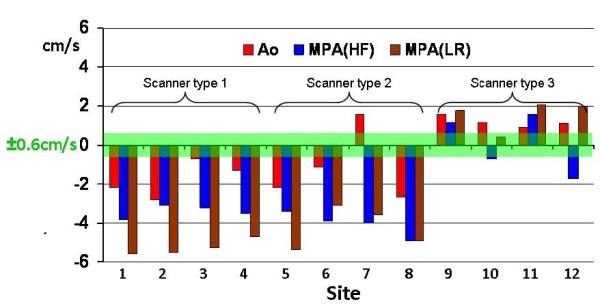
**Uncorrected velocity offset results from all 12 sites**. The largest mean value of the ROI found anywhere up to 5 cm inplane from isocenter for the aortic slice (up to 7 cm for the two MPA slices). The green zone represents the 0.6 cm/s offset described in the Discussion section.

The data of Table 1 is plotted in Figure 5 in comparison with the 0.6 cm/s suggested in the Introduction as an acceptable error level (displayed in green).

Since the MPA plane results appeared larger than the Ao plane on average, the question arises whether this was due to the larger analysis region used (MPA 70 mm vs Ao 50 mm). Analysis was therefore repeated over a central region of up to 50 mm from isocentre for all three plane orientations, giving 1.6 cm/s, 2.2 cm/s and 2.6 cm/s for the Aortic, MPA (head-foot) and MPA (left-right phase-encode) respectively.

## Discussion

### Implications of the study

In 35 of the 36 results, the uncorrected offset exceeded a 0.6 cm/s limit of acceptability which we explained in the Introduction. This implies that all 12 scanners would need to rely on accurate *in-vivo *post-processing correction of velocity offsets for the breath-hold protocol that was used. Referring to the 0.6 cm/s threshold of acceptability defined in the Introduction, the velocity offsets actually measured in the current study were on average *four *times greater than 0.6 cm/s, which could measure the 15% regurgitant fraction example given above as anywhere between 5% and 25%. In some clinical applications, for example in straightforward measurements of peak jet velocity for the assessment of a stenosis, such offsets remain insignificant. But in other situations, the effect may be magnified further, for example if a great vessel is significantly dilated, if stroke volume is reduced, or the R-R interval is increased due to bradycardia.

From Figure 5, it is apparent that all four scanners of type 3 usually showed less severe offsets than most of the data from the others. In spite of the efforts to replicate the imaging parameters on all 3 types, inevitable differences in the gradient waveforms of the three pulse sequences might have caused this difference. It would be unsafe to conclude that scanner type 3 has better eddy-current correction than the other types. The only certain proof would be in a careful comparison of residual eddy currents (i.e. pre-emphasis errors) between scanner types, which would require specialised sequence expertise on each scanner type. To the best of our knowledge this has not been performed.

Velocity offsets caused by Maxwell (concomitant) gradients in phase-contrast imaging are calculable allowing automatic correction during reconstruction [11]. The remaining offset is caused mainly by residual eddy current errors due to small inaccuracies in the pre-emphasis [14] and also any small errors in the concomitant gradient correction. Post-processing to reduce the background offsets was not used in this study for two reasons. First, we wished to see if post-processing was necessary for accurate flow measurements. Second, post-processing might be highly effective in the phantoms used, but could potentially be less effective in-vivo during routine cardiovascular investigation. Of course, there are several other well-recognised sources of error which may compromise the accuracy of breath-hold flow measurements. Examples might arise from reduced spatial or temporal resolution and lower signal to noise ratio using fast acquisition protocols. There may also be physiological effects of breath-holding on flow [15]. However, these are beyond the scope of this study.

### Lessons and Limitations of the study

The methods reported here, which aimed to implement comparable phase contrast acquisitions across CMR sites and systems, turned out to be more challenging than had been anticipated. An initial attempt to invite data collection from different sites resulted in unacceptably wide variations of acquisition parameters, in spite of the careful work undertaken by colleagues at several sites. It became clear that the phantom studies would require pre-set acquisition protocols, transferred digitally between systems. This necessitated cross-system planning and collaboration, which brought to light important variables between systems. Certain variables precluded complete standardisation of acquisition parameters, using clinically available software (For example, the Siemens system did not allow symmetric velocity encoding, whereas the GE system did not allow asymmetric velocity encoding). A further limitation of the study lies in the analysis method of searching for the largest offset in regions displaced in in-plane directions, while the main pulmonary artery tends to have an anterior offset. Taking that example, slices acquired through the isocenter of the magnet may not be optimal for MPA flow measurement. The couch move facility should in this case have been used to relocate the vessel region rather than the centre of the slice. We nevertheless believe that the results recorded give a reasonable indication of the offsets that might be expected in clinical practice.

This study was not designed to find out if any particular plane had greater offsets, but used 45° oblique 'aortic' and 'MPA' planes to test orientations that might typically be used in acquisitions for cardiac output, shunt or regurgitant flow measurement. The apparently larger offset in most MPA planes compared to the aortic planes was not necessarily regarded as representative as orientations might vary in clinical practice, so the significance of this was not tested. Although most of the results have negative polarity, this was not investigated further. Opposite polarities from the same scanner have been reported previously [9].

### Future work

Two obvious questions arise from this work. First, how could a phase-contrast protocol be optimised to minimise the background error in clinical routine? Due to the variability between different scanner types general advice cannot be given beyond the following. Most current CMR systems support automatic couch positioning for flow imaging, aiming to bring the vessel of interest for flow measurement into the isocenter plane (i.e. z = 0 plane, zero head-foot offset) as possible to minimise velocity offset errors. However, implementation of this facility varies between systems and it is important to be aware that the vessel region of interest, not necessarily the centre of the slice, should be positioned with zero offset along the head-foot direction. (For the ascending aorta, transverse imaging can solve this difficulty). Beyond this basic step, any further optimisation of sequence waveforms to minimise background offsets is highly sensitive to residual errors in the correction of eddy currents. The term "residual errors" refers to the difference between the eddy current-related field distortion and the pre-calibrated compensation for eddy-currents (known as "pre-emphasis") applied to the gradient waveforms. The accumulation of this residual phase error during the time between velocity encoding and the echo may be positive or negative, leading to positive or negative offsets in the phase-subtraction velocity image. It can be estimated that a velocity offset of 0.6 cm/s in a 200 cm/s VENC scan may arise from a residual error (of time-constant ≈ Te) of ≈ 0.01% of the amplitude of the gradient change made for velocity-encoding, which is extremely challenging for manufacturers to achieve. This is around ten times more demanding of accurate pre-emphasis than balanced SSFP cine imaging, and it remains uncertain whether this could ever be improved reliably so that offset post-processing correction techniques will not be needed.

The second question that arises from the work is, how reliable are velocity offset correction techniques *in-vivo *since it appears that most systems require them? This study did not use any correction methods which might have appeared unrealistically effective when applied to images from the large uniform, gelatin phantoms. Correction software may be less reliable *in-vivo*, particularly if there is insufficient stationary tissue in the acquisition plane, or if signal from it is poor. Furthermore, phase-encode wraparound and possible spatial non-linearity of the offset may be problematic. An alternative but more time-consuming approach to offset correction requires identical flow acquisitions using a stationary phantom after a patient study, subtracting the corresponding apparent phantom velocities from the clinical acquisition [9] (with smoothing to avoid SNR reduction by the subtraction). This approach should correct all background offsets precisely, even if non-linearly distributed, provided that the offsets are stable as a function of time. More work is required to evaluate the *in-vivo *reliability of correction methods, for example using post-acquisition phantom scans as a temporary gold standard [10].

For use in clinical routine, the results imply that sites should be cautious about potential inaccuracy unless they have proved otherwise for the protocols and correction methods used there.

In conclusion, all of the 12 systems tested (without offset correction methods) showed velocity offset values larger than the 0.6 cm/s shown necessary for <10% error in the most sensitive cardiac applications. It is therefore necessary to have a reliable background offset correction method for images acquired by a typical breath-hold flow protocol. The reliability of background velocity offset correction techniques needs to be tested with respect to clinical flow acquisitions.

## Potentially Competing interests

Peter Gatehouse: Research agreement with Siemens.

David Firmin: Research agreement with Siemens.

John Totman: Research agreement with Philips.

Martin Graves: Research support from GE.

All other authors: None.

## Authors' contributions

PJK, JS, AvR, JS-M, ML forming the EuroCMR Working Group of the European Society of Cardiology (ESC) conceived the study. PG designed the study with the assistance of MPR, MH, MG, JT. All other authors installed the designated test protocol, made appropriate test phantoms and ran the tests.
